# National trends in the proportion of in-hospital deaths by cause of death among older adults with long-term care: a nationwide observational study in Japan from 2007 to 2017

**DOI:** 10.1186/s12877-021-02700-1

**Published:** 2022-01-03

**Authors:** Yuta Taniguchi, Masao Iwagami, Xueying Jin, Nobuo Sakata, Mikiya Sato, Taeko Watanabe, Kyoko Hanari, Kazuhiro Abe, Haruko Noguchi, Nanako Tamiya

**Affiliations:** 1 Graduate School of Comprehensive Human Sciences, University of Tsukuba, Ibaraki, Japan; 2 Health Services Research and Development Center, University of Tsukuba, Ibaraki, Japan; 3 Department of Health Services Research, Faculty of Medicine, University of Tsukuba, 1-1-1 Tenno-dai, Tsukuba, Ibaraki, 305-8575 Japan; 4 Sumitomo Heavy Industries, Ltd., Human Resources Group, Health Services Center, Tokyo, Japan; 5 Department of Public Health, Graduate School of Medicine, The University of Tokyo, Tokyo, Japan; 6 Takemi Program in International Health, Harvard T.H.Chan School of Public Health, Boston, MA USA; 7 Faculty of Political Science and Economics, Waseda University, Tokyo, Japan

**Keywords:** End-of-life care, Place of death, Health services research, Japan, Long-term care

## Abstract

**Background:**

Japan has promoted end-of-life care at home and in long-term care facilities, and the total proportion of in-hospital deaths has decreased recently. However, the difference in trends of in-hospital deaths by the cause of death remains unclear. We investigated the variation in trends of in-hospital deaths among older adults with long-term care from 2007 to 2017, by cause of death and place of care.

**Methods:**

Using the national long-term care insurance registry, long-term care claims data, and national death records, we identified people aged 65 years or older who died between 2007 and 2017 and used long-term care services in the month before death. Using a joinpoint regression model, we evaluated time trends of the proportion of in-hospital deaths by cause of death (cancer, heart diseases, cerebrovascular diseases, pneumonia, and senility) and place of care (home, long-term care health facility, or long-term care welfare facility).

**Results:**

Of the 3,261,839 participants, the mean age was 87.0 ± 8.0 years, and 59.2% were female. Overall, the proportion of in-hospital deaths decreased from 66.2% in 2007 to 55.3% in 2017. By cause of death, the proportion of in-hospital deaths remained the highest for pneumonia (81.6% in 2007 and 77.2% in 2017) and lowest for senility (25.5% in 2007 and 20.0% in 2017) in all types of places of care. The joinpoint regression analysis showed the steepest decline among those who died of senility, especially among long-term care health facility residents.

**Conclusions:**

The findings of this nationwide study suggest that there was a decreasing trend of in-hospital deaths among older adults, although the speed of decline and absolute values varied widely depending on the cause of death and place of care.

**Supplementary Information:**

The online version contains supplementary material available at 10.1186/s12877-021-02700-1.

## Background

There is a rise in the proportion of older adult populations worldwide due to decline in fertility rates and extension of life expectancy [1]. This makes where to provide end-of-life care a matter of global interest. Japan is facing a rapid aging of the population and has the highest proportion of older adults in the world [1]. The Japanese government introduced a long-term care insurance system in 2000 to cover the costs for long-term care of people aged 65 or older, and people aged 40–64 with specific diseases [2].

As the population is aging, the number of deaths is also increasing. In 2017, 73% of the deaths in Japan occurred in hospitals [3], which was higher than that in other countries such as the United States (29.8%), England (46.0%), or Canada (59.9%) [4]. Meanwhile, 55% of the general population in Japan hope to die at home [5]. Also, the number of deaths in Japan is expected to increase from 1.3 million in 2017 to 1.6 million by 2030 [3, 6], and hospital bed capacity as a place of death is predicted to be in short supply [7, 8]. To fill this gap, the Japanese government has, since 2006, promoted end-of-life care at home and at long-term care (LTC) facilities, by increasing the financial incentives for service providers who attend to patients’ deaths at home and at LTC facilities [9–11].

Overall, it has been reported that after peaking at 79.8% in 2005, the proportion of in-hospital deaths has decreased gradually to 73.0% in 2017 [3]. Moreover, a previous study revealed a decreasing trend of in-hospital deaths among older adults using LTC services, with a sharper reduction among those in a residential aged care facility than in the community, possibly due to the incentivization of end-of-life care outside hospitals [12]. A few other studies on LTC welfare facility residents [13] and patients with dementia [14] also speculated that the declining trends of in-hospital deaths may be attributable to the promotion of end-of-life care at home and in LTC facilities. However, these previous studies did not evaluate differences in trends of in-hospital deaths between the causes of death. To promote end-of-life care at home and in LTC facilities, more detailed information would be helpful for planning cause-and-place-specific strategies, as the use and needs of services can differ between diseases. Therefore, this descriptive study aimed to evaluate the difference in transition of in-hospital deaths among older adults in LTC between 2007 and 2017, by cause of death in different types of places of care.

## Methods

### Data source

We obtained data for the period 2007 to 2017, from the national long-term care insurance (LTCI) registry, LTCI claims data and national death records, with permission from the Ministry of Health, Labour, and Welfare under the Statistics Act, Article 33. First, we identified older adults (≥ 65 years) who were withdrawn from the LTCI registry and linked them to national death records, using the following four variables: insurer code (city code), sex, birth year and month, and disqualification date (i.e., moving out or death). We excluded individuals who were not uniquely identifiable with these four variables. Second, using LTCI claims data, we identified individuals who used the LTCI services in the month before death, with their place of care (i.e., home, LTC health facilities, or LTC welfare facilities).

Using the 10th revision of the International Statistical Classification of Diseases (ICD-10) codes, we identified individuals who died of the following major causes of death in Japan [3]: cancer (C00–C97), heart diseases (I01-I02, I05-I09, I20-I25, I27, I30-I51), cerebrovascular diseases (I60-I69), pneumonia (J12-J18), and senility (R54).

### Study population

The inclusion criteria of the study were i) individuals aged 65 or older, ii) those who died between 2007 and 2017, iii) those with care need level 1 or above, and iv) those who used LTC services at home or LTC facilities in the month before death. Under the LTC insurance system in Japan, eligibility for LTC services is assessed and categorized into seven levels: support level 1 (least disabled), 2 and care need levels 1–5 (most disabled) [2]. We included persons with care need level 1 or above because individuals with support levels 1–2 have limited eligibility for LTC facility services [15, 16]. We categorized the study population into three groups according to the place of care in the month before death: home, LTC health facilities (also called geriatric health services facilities, or *roujinhokenshisetsu* or “*roken*” in Japanese), and LTC welfare facilities (also called special nursing homes, or *tokubetsuyougoroujinhoumu* or “*tokuyo*” in Japanese). The LTC health facility is a rehabilitation facility that is used for short periods and employs a full-time physician [9]. The LTC welfare facility is a daily life facility providing constant care services, where a part-time physician is permissible [9]. Also, since 2006, both the LTC health facilities and welfare facilities have been encouraged to provide end-of-life care by financial incentives from the Japanese government [9–11].

The exclusion criteria were i) those living in group homes for older adults with dementia and geriatric apartments and ii) few patients who lived at multiple types of sites in the month before death. Group homes for older adults with dementia and geriatric apartments provide residents with services such as housework help or long-term care, and their characteristics as places of care are different from one’s own home [9].

### Statistical analysis

We calculated the proportion of in-hospital deaths for each year from 2007 to 2017, as the number of people who died in a hospital divided by the number of total deaths, overall (any hospital death) and by the cause of death (cancer, heart diseases, cerebrovascular diseases, pneumonia, and senility) in each place of care (home, LTC health facility, or LTC welfare facility).

To evaluate the time trends of the proportion of in-hospital deaths, we fitted the joinpoint regression model for each cause of death in each place of care. In the joinpoint regression model, we determined the trend change points (joinpoint), estimated the annual percentage change between joinpoints, and calculated the average annual percentage change [17]. To investigate the trend difference by care need levels, we repeated the analysis by care need levels (care need level 1–2 [low to moderate need] or care need level 3–5 [moderate to severe need]). *P*-values < 0.05 were considered statistically significant.

We used Stata 15 (StataCorp, TX, USA), Microsoft Excel for Mac 16.33 (Microsoft, WA, USA), and Joinpoint Regression Program 4.9.0.0 (National Cancer Institute, MD, USA) for analysis.

### Additional analysis

Since the age distribution may have changed over the decade, we conducted additional analyses using a logistic regression model adjusting for age. We calculated the adjusted odds ratios of in-hospital death for each cause of death at each place of care in each year from 2008 to 2017 (reference: 2007).

### Ethics approval

Because of the anonymized nature of the data, individual participants’ consent was waived. The study was approved by the Ethics Committee of the University of Tsukuba (approval no. 1324–2).

## Results

From the 6,632,539 older adults who were eligible from the LTCI registry during the period between 2007 and 2017, we uniquely identified 6,426,937 people (96.9%) using the four variables (insurer city code, sex, birth year and month, and disqualification date), and successfully linked 5,722,084 people (86.3%) with the national death records. Of this population, 3,261,839 utilized LTC services in the month before death with care need level 1 or higher. These included 1,947,489 people at home, 383,522 at the LTC health facility, and 588,838 at the LTC welfare facility. The 24,079 patients who received LTCI services at multiple sites (home, LTC health facility, or LTC welfare facility) in the month before death were excluded in the analysis. The number of final study participants was 2,919,849. Table 1 shows the characteristics of the participants. The mean age at death was 87.0 ± 8.0 years and 59.2% were female.

**Table 1:** Characteristics of older adults who used long-term care services in the month before death

	Place of care in the month before death		
	Home	LTC health facility	LTC welfare facility
No	1,947,489	383,522	588,838
Age, years, Mean (SD)	85.8 (8.1)	88.9 (7.2)	89.8 (7.2)
Female sex, No (%)	1,039,644 (53.4)	251,900 (65.7)	436,990 (74.2)
Cause of death, No (%)			
Cancer	507,275 (26.0)	37,179 (9.7)	41,548 (7.1)
Heart diseases	332,048 (17.1)	67,630 (17.6)	91,376 (15.5)
Cerebrovascular diseases	157,555 (8.1)	53,267 (13.9)	79,648 (13.5)
Pneumonia	198,867 (10.2)	59,566 (15.5)	86,883 (14.8)
Senility	154,420 (7.9)	51,149 (13.3)	114,948 (19.5)
The others	597,324 (30.7)	114,731 (29.9)	174,435 (29.6)
Year of death			
2007	127,741	26,003	38,081
2008	140,520	27,991	42,630
2009	142,469	28,517	43,404
2010	154,507	30,801	46,317
2011	166,153	33,214	50,407
2012	182,448	36,049	54,907
2013	185,934	38,022	57,883
2014	195,295	38,875	58,942
2015	218,885	38,169	58,588
2016	211,707	42,313	66,462
2017	221,830	43,568	71,217

*LTC* long-term care, *SD* standard deviation

The overall proportion of in-hospital deaths decreased from 66.2% in 2007 to 55.3% in 2017. Between 2007 and 2017, the percentage of in-hospital deaths declined, by cause of death, from 70.0 to 59.4% for cancer (*n* = 586,002), from 64.3 to 60.1% for heart diseases (*n* = 491,054), from 61.5 to 51.2% for cerebrovascular diseases (*n* = 290,470), from 81.6 to 77.2% for pneumonia (*n* = 345,316), and from 25.5 to 20.0% for senility (*n* = 320,517).

**Fig. 1:**
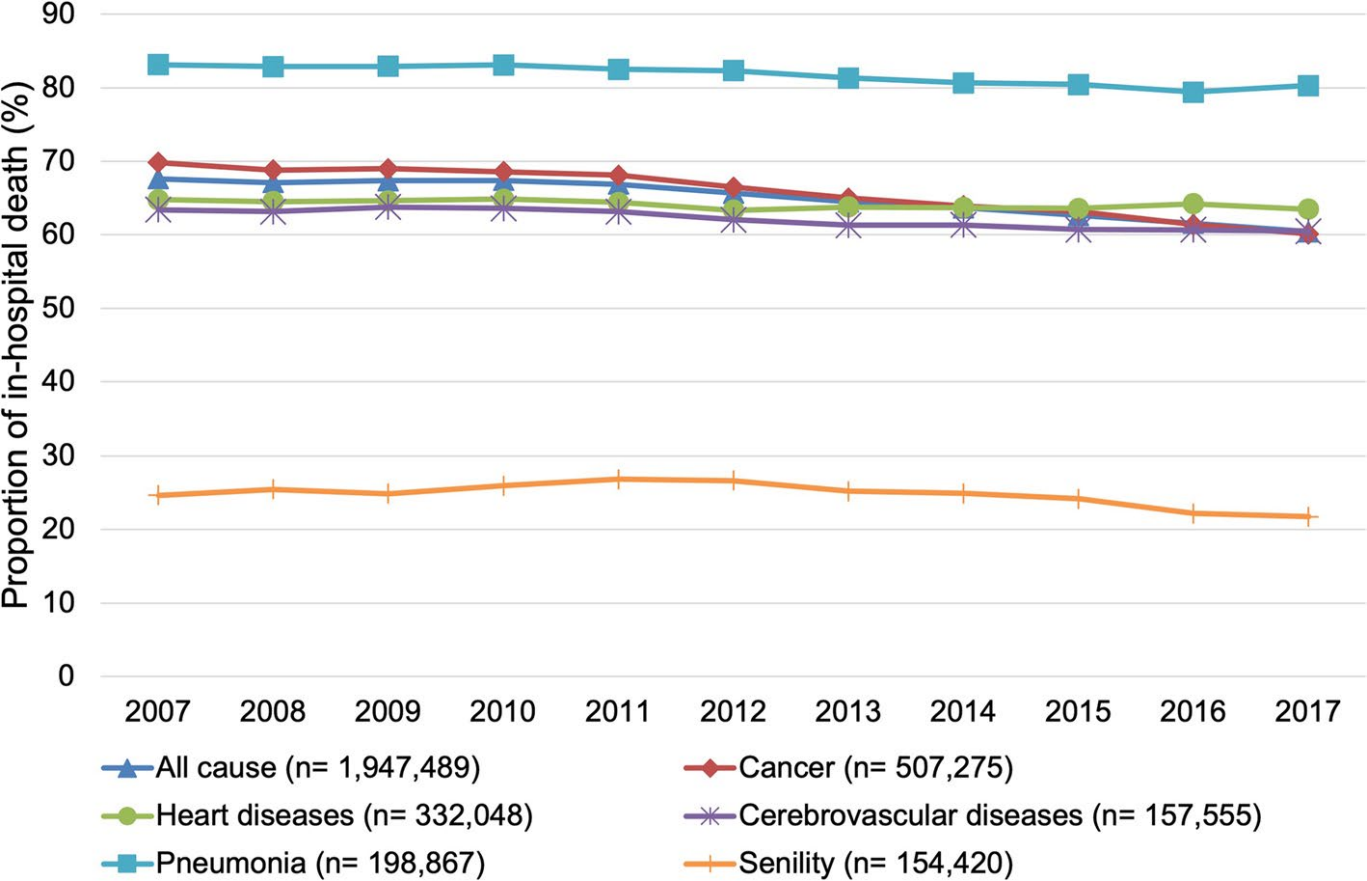
Trends of the proportion of in-hospital deaths of those living at home in the month before death (*n* = 1,947,489)

**Fig. 2:**
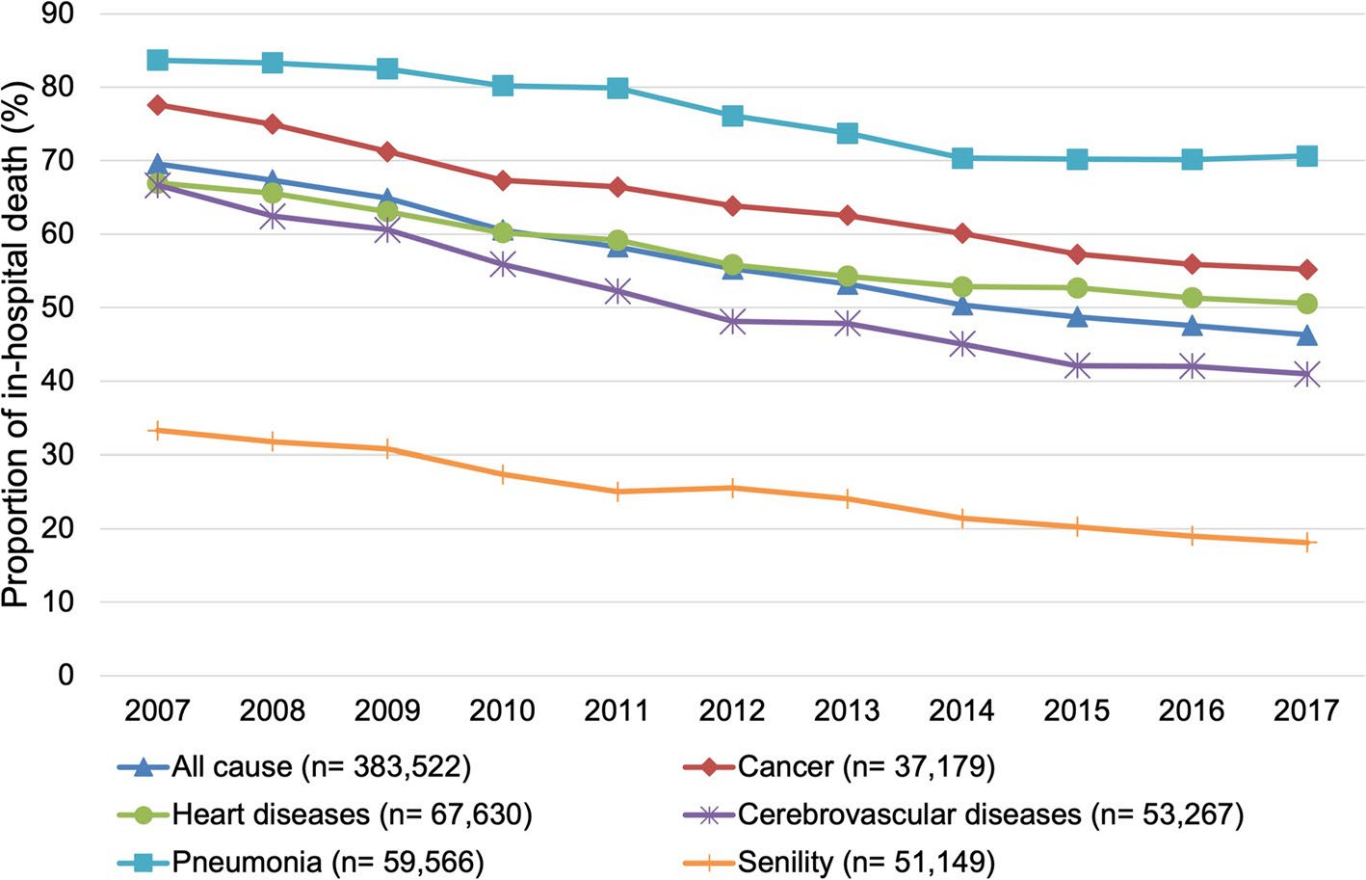
Trends of the proportion of in-hospital deaths of long-term care health facility residents in the month before death (*n* = 383,522)

**Fig. 3:**
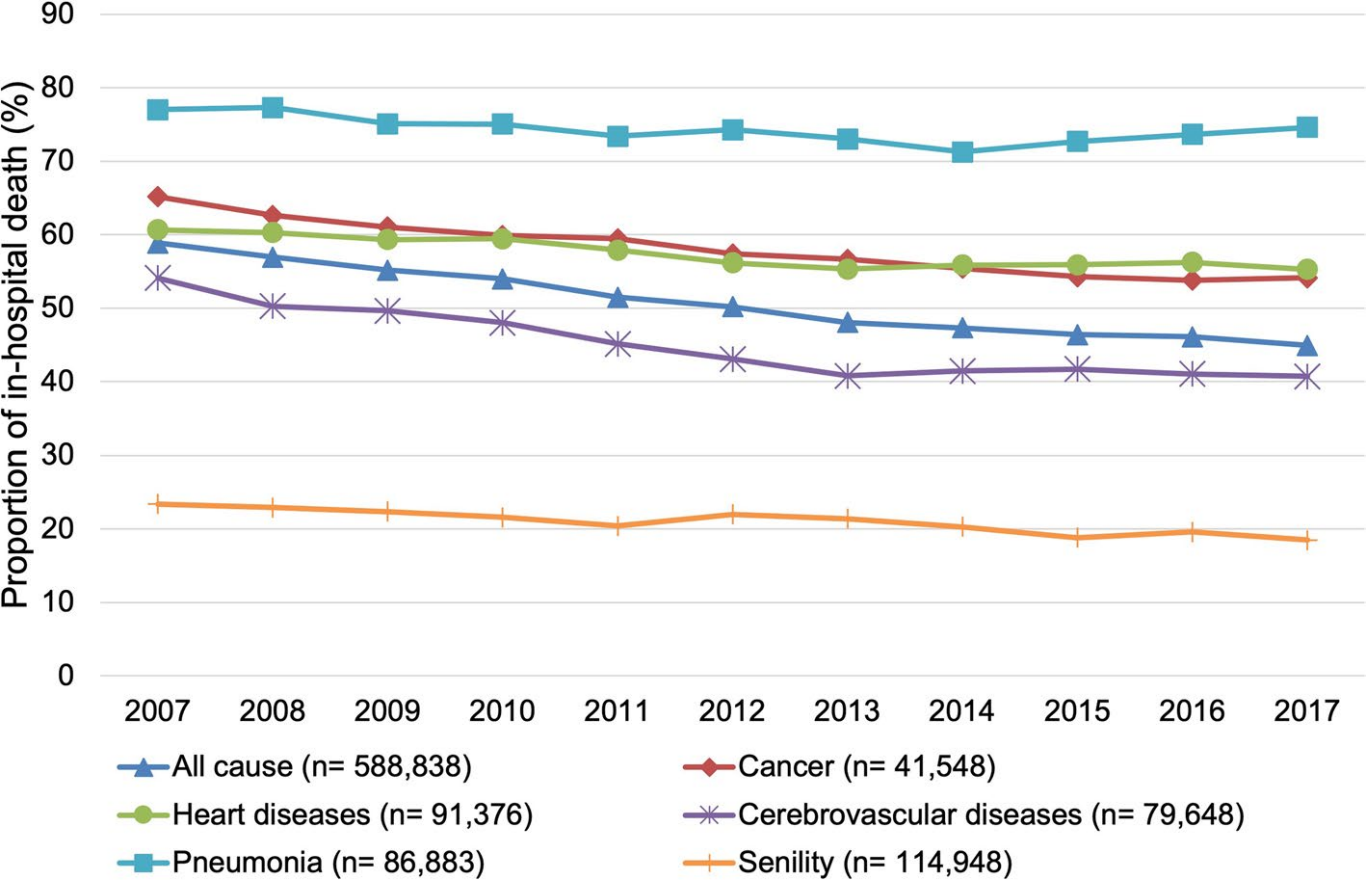
Trends of the proportion of in-hospital deaths of long-term care welfare facility residents in the month before death (*n* = 588,838)

The trends of in-hospital deaths by cause of death and place of care are presented in Figs. 1, 2, 3. The proportion of in-hospital deaths remained the highest for pneumonia and lowest for senility in all three types of places of care. The joinpoint regression analysis showed that the average annual percentage change was statistically significant for all causes of death in all types of places of care, except for the LTC welfare facility residents who died of pneumonia (Table 2). The declining trend for each cause of death was the sharpest among the LTC health facility residents, followed by the LTC welfare facility residents or those residing at home. The average annual percentage decrease was largest for the LTC health facility residents who died of senility (6.1%).

**Table 2:** Trends of the proportion of in-hospital deaths, by cause of death and place of care

		Trend1		Trend2			
Place of care	Cause of death	Years	APC (%)	Years	APC (%)	Average APC	[95% CI]
Home	All cause	2007–2011	− 0.2	2011–2017	−1.7*	−1.1*	[−1.2 to− 1.0]
	Cancer	2007–2011	−0.6*	2011–2017	−2.0*	−1.4*	[−1.6 to− 1.2]
	Heart diseases	2007–2017	−0.2*			−0.2*	[−0.3 to− 0.1]
	Cerebrovascular diseases	2007–2017	−0.6*			−0.6*	[−0.8 to− 0.4]
	Pneumonia	2007–2017	−0.5*			−0.5*	[−0.6 to− 0.3]
	Senility	2007–2012	1.6*	2012–2017	−4.1*	−1.3*	[−2.2 to−0.4]
LTC health facility	All cause	2007–2014	− 4.6*	2014–2017	−2.9*	−4.1*	[−4.5 to−3.7]
	Cancer	2007–2017	− 3.4*			−3.4*	[− 3.7 to− 3.1]
	Heart diseases	2007–2013	−3.6*	2013–2017	−1.8*	−2.9*	[−3.3 to− 2.4]
	Cerebrovascular diseases	2007–2015	−5.5*	2015–2017	−1.1	−4.7*	[−5.8 to−3.5]
	Pneumonia	2007–2017	−2.1*			−2.1*	[−2.6 to−1.7]
	Senility	2007–2017	−6.1*			−6.1*	[−6.6 to−5.5]
LTC welfare facility	All cause	2007–2013	−3.3*	2013–2017	−1.7*	−2.7*	[−2.9 to− 2.4]
	Cancer	2007–2015	− 2.1*	2015–2017	−0.1	−1.7*	[−2.2 to− 1.2]
	Heart diseases	2007–2013	−1.5*	2013–2017	−0.0	− 0.9*	[−1.4 to− 0.5]
	Cerebrovascular diseases	2007–2013	−4.3*	2013–2017	−0.2	−2.7*	[−3.3 to− 2.1]
	Pneumonia	2007–2014	−1.1*	2014–2017	1.3	−0.4	[− 0.8 to 0.1]
	Senility	2007–2017	−2.2*			−2.2*	[−2.8 to−1.5]

* Significantly different from zero (*p* < 0.05). *LTC* long-term care, *APC* annual percent change

The results of the analyses by care need levels are shown in the additional files (Additional Figs. 1-2 and Additional Table 1). The results were similar, and the proportion of in-hospital deaths remained the highest for pneumonia and lowest for senility regardless of the care need level. People with higher care need levels showed a steeper decline in in-hospital deaths for every cause of death. The additional analyses with the logistic regression models adjusting for age showed similar trends (Additional Figs. 3, 4, 5).

## Discussion

This study, to the best of our knowledge, is the first to investigate the difference in the transition of the proportion of in-hospital deaths among older adults in LTC by cause of death and place of care. From 2007 to 2017, there has been a decreasing trend in in-hospital deaths among older adults regardless of the cause of death. The speed of the decline and absolute values varied widely between the cause of death and place of care, possibly due to the different prognosis for each disease.

By cause of death, the proportion of in-hospital deaths remained highest for pneumonia throughout the study period and lowest for senility in all three types of places of care. A questionnaire survey in 241 LTC welfare facilities reported that not having pneumonia as the cause of death was positively associated with dying at LTC welfare facilities [13]. Our study showed comparable results at the national level, not only for the LTC welfare facility but also for the LTC health facility and at home. We assume that patients with pneumonia have a higher possibility of recovery than those with other conditions such as senility, and this may have led to their transfer to hospital even at the terminal stage, and consequently in-hospital deaths. Conversely, the decreasing trend of in-hospital deaths was sharpest among patients with senilty, especially among LTC health facility residents. This may suggest an improvement in end-of-life care among patients with senility, especially at LTC health facilities.

Not only for senility but also for every cause of death, we found the steepest decline in in-hospital deaths among LTC health facility residents, followed by LTC welfare facility residents or those living at home. This finding is consistent with that of a previous study that reported a sharper reduction in in-hospital deaths among those at the LTC facilities than in the community [12]. However, our study further differentiated LTC health facility and LTC welfare facility to reveal more details, in that the declining trend was steeper among LTC health facility residents than among LTC welfare facilities. This variation may have been caused by the difference in accessible medical care in each place of care. Attending death would be more feasible at the LTC health facility, which employs a full-time physician, than an LTC welfare facility, where often only a part-time physician is available. In addition, the Japanese government added payment for the doctor’s visiting the LTC health facilities at night and on holidays since 2008 [18], which may have encouraged medical treatment at the LTC health facility. Additionally, we considered the possible influence of financial incentives for LTC health facilities treating pneumonia which were introduced in 2012, with the aim of reducing the transfer of patients with pneumonia to the hospital [19]. However, we did not observe a statistically significant change in the decreasing trend before and after the introduction of the incentive in 2012, among those who died of pneumonia at the LTC health facility.

Finally, by care need levels, we observed a steeper decreasing trend in in-hospital deaths among those with higher care need levels. This is consistent with findings of previous studies which indicated that a higher care need level or a poorer functional status was negatively associated with in-hospital deaths [12, 20]. As the level of care needs deteriorated, the five-year survival rates showed a decline, from 72.3% for people with the least disability support level, to 22.2% for those with care need level 5 (most disabled) [21]. The most common occasion for health professionals to confirm patients’ intentions about their desired medical care near death, was when patients were close to death [22]. Therefore, older adults with higher care need levels, who had a shorter prognosis, may have had more chance to make their desire for end-of-life care known, which could have resulted in the fewer in-hospital deaths. Promotion of patients’ self-determination at the end of life in the recent years by the Japanese government [23], might have led to steeper decreasing trends in in-hospital deaths, especially among those with higher care need levels.

This study has several limitations. First, we could not confirm a causal relationship between the health policy to promote end-of-life care at home or LTC facilities and the declining trends of in-hospital death. Second, we were unable to consider underlying diseases other than the cause of death due to lack of information. In addition, the new coding rule of the underlying cause of death was implemented in 2017 according to the update of ICD-10 in 2013 [24]. Therefore, the patient’s disease condition and the cause of death may have been different prior to and after 2017. Third, we could not link the LTCI registry to national death records for a few participants who were not uniquely identified with available variables. Finally, while we have discussed the differences between available medical resources among the three types of places of care that may explain the variation in the proportion of in-hospital deaths, we did not consider the individual characteristics of each patient or LTC facility; these might have affected the proportion of in-hospital deaths.

## Conclusions

In conclusion, among older adults who lived at home or in a LTC facility in the month before death, the proportion of in-hospital deaths decreased between 2007 and 2017, and it remained the highest for pneumonia and the lowest for senility. The decreasing trend was observed to be greater in senile patients, especially among the LTC health facility residents, which may suggest an improvement in end-of-life care at the LTC health facilities. To expand the end-of-life care at home and at LTC facilities, the assessment and enhancement of care appropriate for each disease could be effective. Moreover, since Japan has the highest proportion of older people globally, our results could be informative for the other countries experiencing an increasingly aged population.

## Supplementary Information

**Additional file 1: Additional Figure 1.** Trends of the proportion of in-hospital deaths of those in care need levels 1-2 in the month before death (*n*= 729,847). **Additional Figure 2.** Trends of the proportion of in-hospital deaths of those in care need levels 3-5 in the month before death (*n*= 2,190,002). **Additional Figure 3.** Age-adjusted odds ratios for in-hospital deaths of those living at home in the month before death (*n*= 1,947,489). **Additional Figure 4.** Age-adjusted odds ratios for in-hospital deaths of long-term care health facility residents in the month before death (*n*= 383,522). **Additional Figure 5.** Age-adjusted odds ratios for in-hospital deaths of long-term care welfare facility residents in the month before death (*n*= 588,838).

**Additional file 2: Additional Table 1.** Trends of the proportion of in-hospital deaths, by cause of death and care need levels.

## Data Availability

The datasets generated and/or analyzed during the current study are not publicly available because the Japanese Ministry of Health, Labour and Welfare owns the original data and only approved the secondary use of the data for the current study.

## Abbreviations

ICD-10: International Classification of Diseases, Tenth Revision
LTC: Long-term care
LTCI: Long-term Care Insurance
