# Supplementary material for: National trends in the proportion of in-hospital deaths by cause of death among older adults with long-term care: a nationwide observational study in Japan from 2007 to 2017
Source: BMC Geriatr. 2022 Jan 3;22:6. doi: 10.1186/s12877-021-02700-1 (PMC8722128; doi:10.1186/s12877-021-02700-1)

Taniguchi et al.

Additional Figure 1. Trends of the proportion of in-hospital deaths of those in care need levels 1–2 in the month before death (n= 729,847)

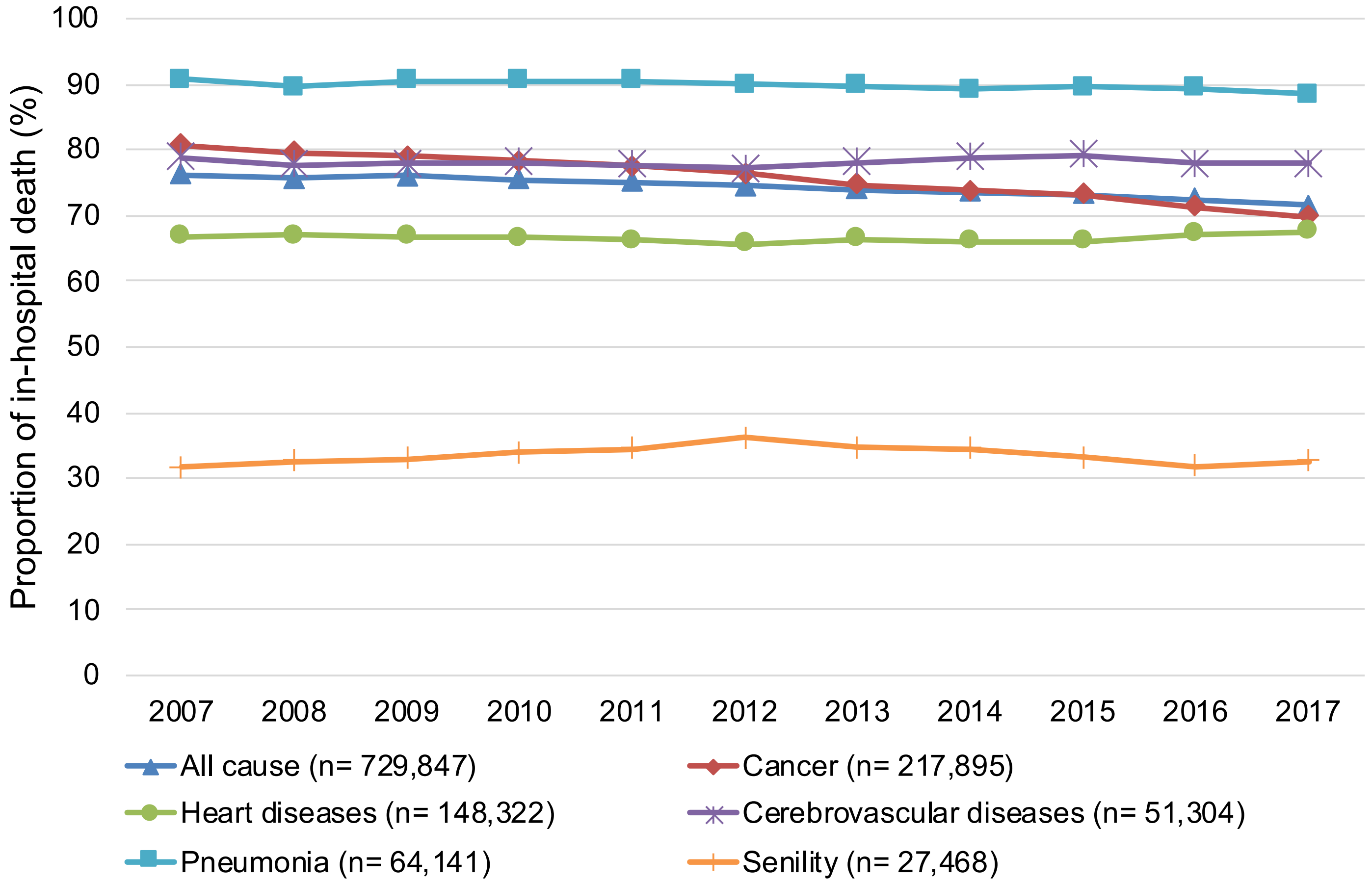

Taniguchi et al.

Additional Figure 2. Trends of the proportion of in-hospital deaths of those in care need levels 3–5 in the month before death (n= 2,190,002)

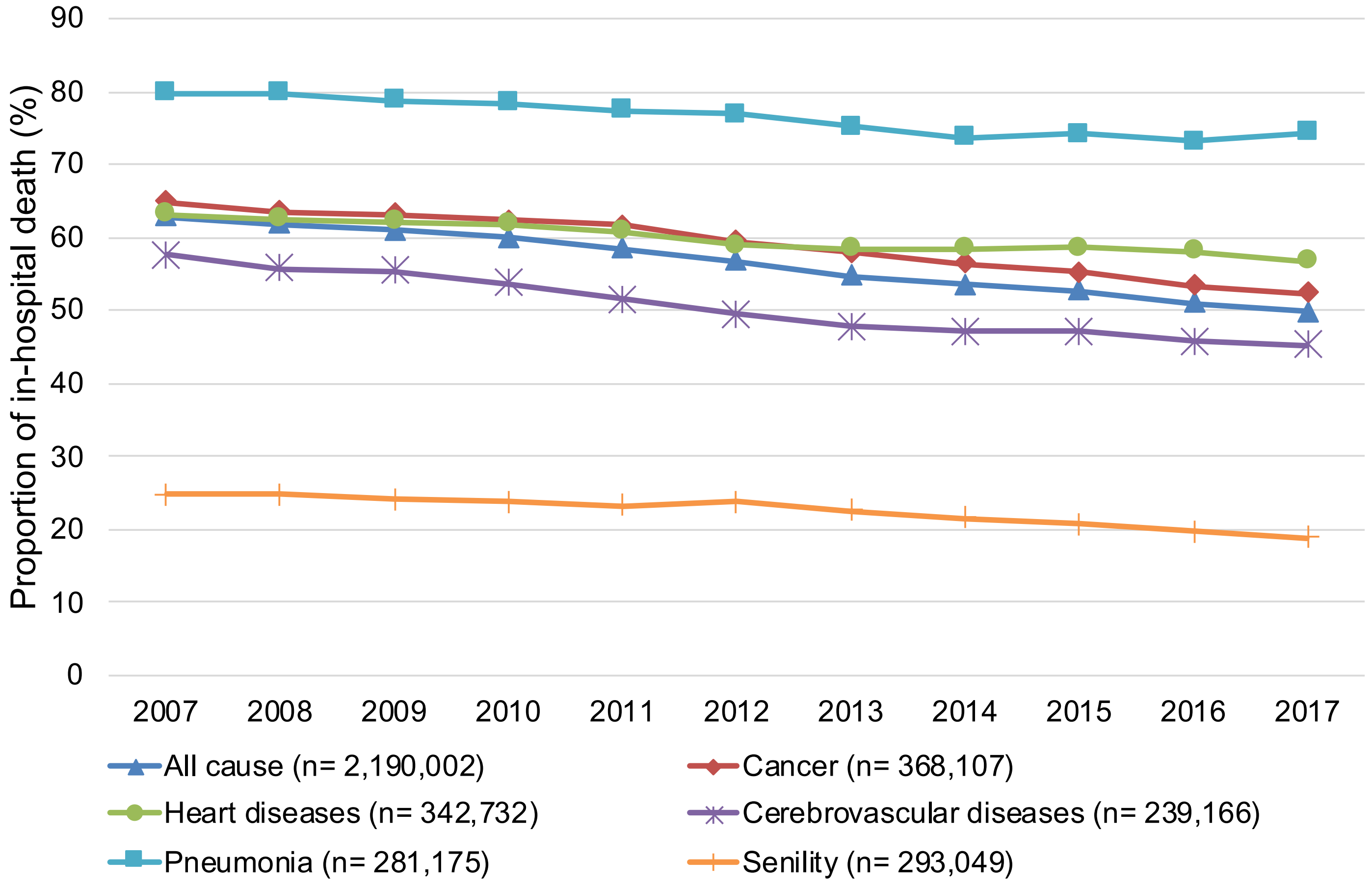

Taniguchi et al.

Additional Figure 3. Age-adjusted odds ratios for in-hospital deaths of those living at home in the month before death (n= 1,947,489)

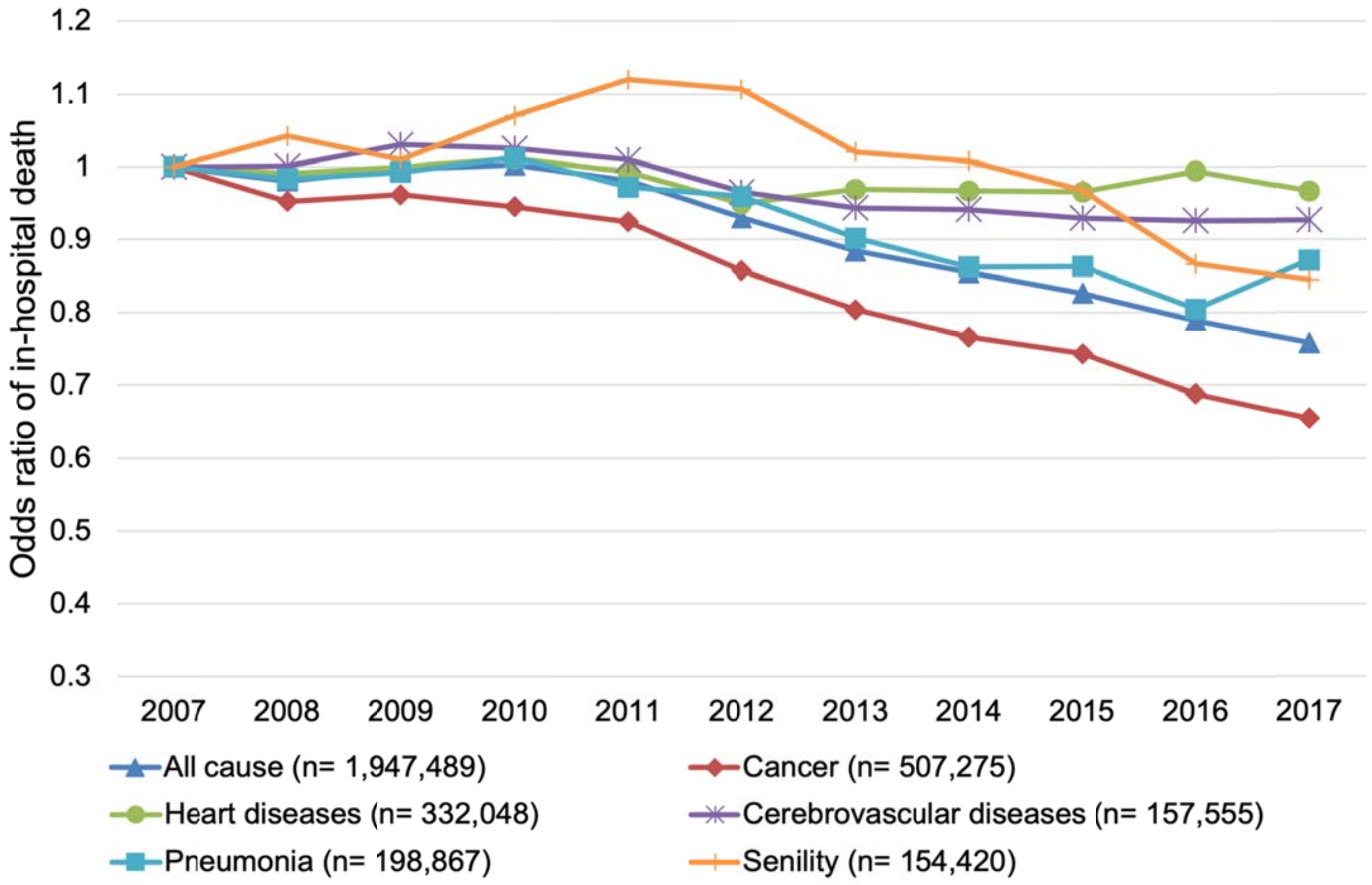

Taniguchi et al.

Additional Figure 4. Age-adjusted odds ratios for in-hospital deaths of long-term care health facility residents in the month before death (n= 383,522)

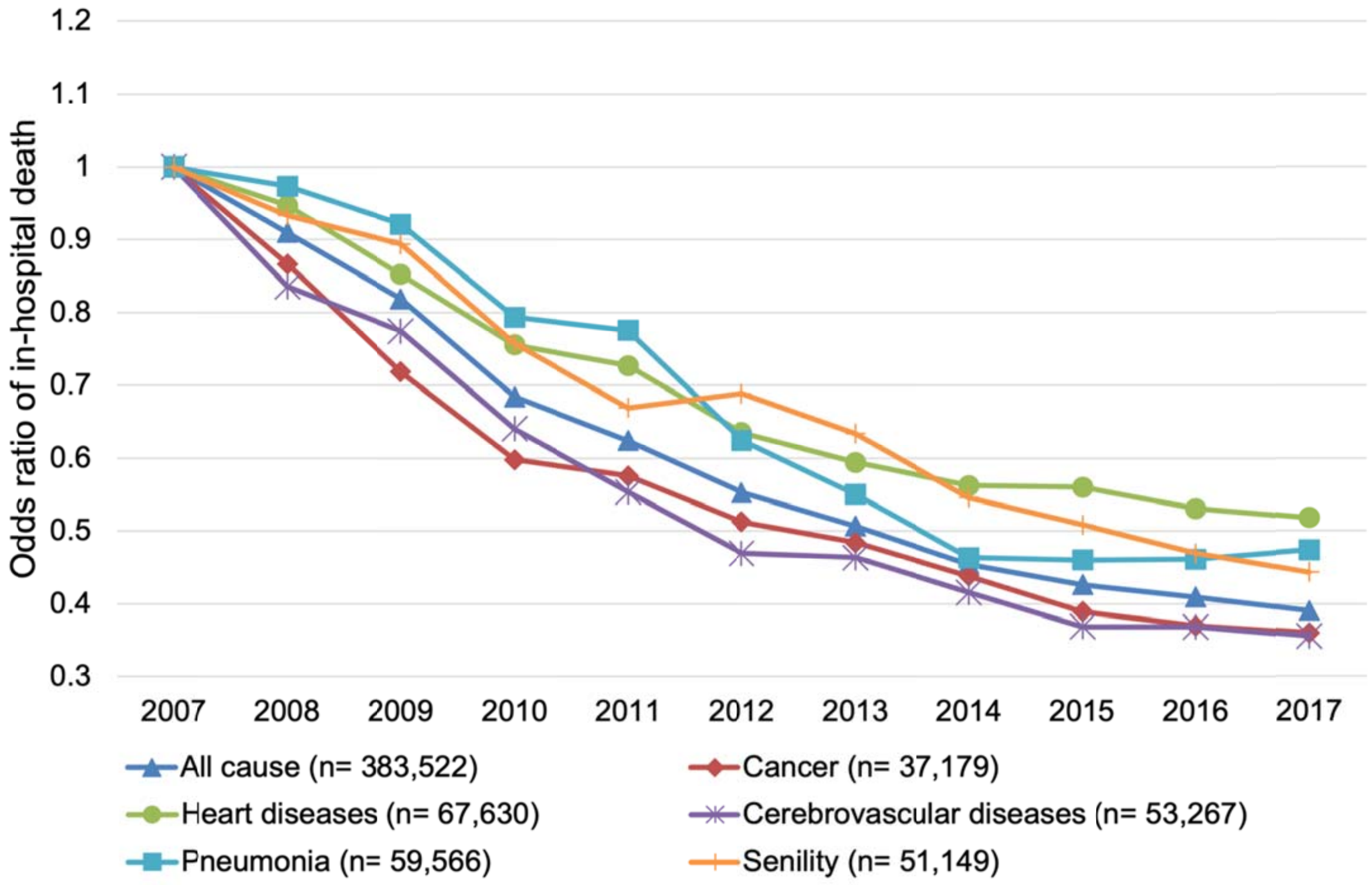

Taniguchi et al.

Additional Figure 5. Age-adjusted odds ratios for in-hospital deaths of long-term care welfare facility residents in the month before death (n= 588,838)

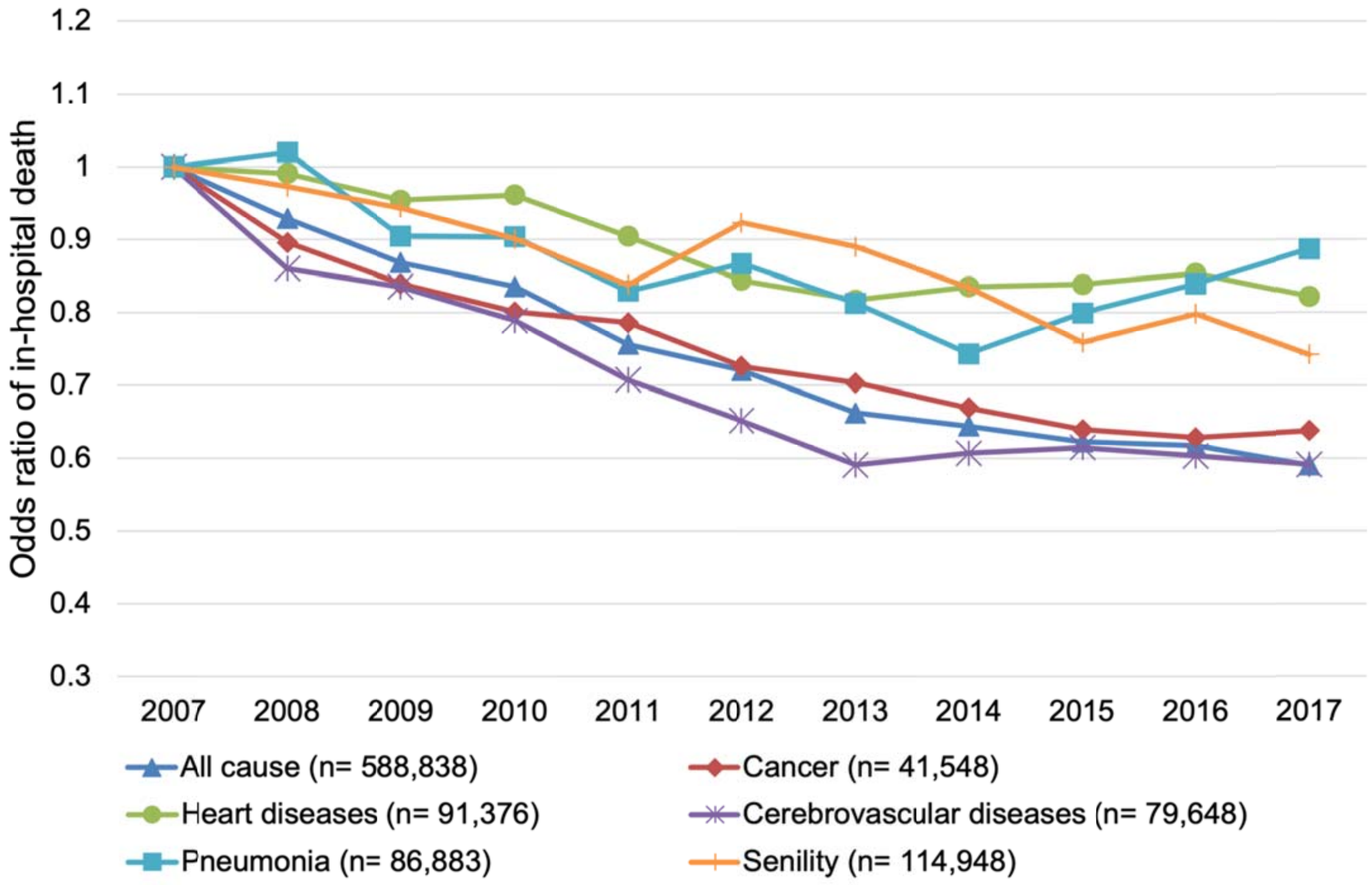

Supplement: Supplementary file 1 — Additional file 1: Additional Figure 1. Trends of the proportion of in-hospital deaths of those in care need levels 1-2 in the month before death (n= 729,847). Additional Figure 2. Trends of the proportion of in-hospital deaths of those in care need levels 3-5 in the month before death (n= 2,190,002). Additional Figure 3. Age-adjusted odds ratios for in-hospital deaths of those living at home in the month before death (n= 1,947,489). Additional Figure 4. Age-adjusted odds ratios for in-hospital deaths of long-term care health facility residents in the month before death (n= 383,522). Additional Figure 5. Age-adjusted odds ratios for in-hospital deaths of long-term care welfare facility residents in the month before death (n= 588,838). [file 12877_2021_2700_MOESM1_ESM.pdf]
