# Supplementary material for: National trends in the proportion of in-hospital deaths by cause of death among older adults with long-term care: a nationwide observational study in Japan from 2007 to 2017
Source: BMC Geriatr. 2022 Jan 3;22:6. doi: 10.1186/s12877-021-02700-1 (PMC8722128; doi:10.1186/s12877-021-02700-1)
Supplement: Supplementary file 2 — Additional file 2: Additional Table 1. Trends of the proportion of in-hospital deaths, by cause of death and care need levels. [file 12877_2021_2700_MOESM2_ESM.pdf]

Taniguchi et al.

Additional Table 1. Trends of the proportion of in-hospital deaths, by cause of death and care need levels

| | | Trend 1 | | Trend 2 | | | |
|---|---|---|---|---|---|---|---|
| Care need levels | Cause of death | Years | APC (%) | Years | APC (%) | Average APC | [95% CI] |
| Care need levels 1-2 | All cause | 2007-2011 | −0.3 | 2011-2017 | −0.8* | −0.6* | [−0.7 to −0.4] |
| | Cancer | 2007-2011 | −0.9* | 2011-2017 | −1.7* | −1.4* | [−1.6 to −1.2] |
| | Heart diseases | 2007-2014 | −0.2* | 2014-2017 | 0.9* | 0.1 | [−0.1 to 0.3] |
| | Cerebrovascular diseases | 2007-2017 | −0.0 | | | −0.0 | [−0.2 to 0.2] |
| | Pneumonia | 2007-2017 | −0.2* | | | −0.2* | [−0.3 to −0.1] |
| | Senility | 2007-2012 | 2.4* | 2012-2017 | −2.2* | 0.1 | [−0.6 to 0.8] |
| Care need levels 3-5 | All cause | 2007-2010 | −1.6* | 2010-2017 | −2.6* | −2.3* | [−2.6 to −2.1] |
| | Cancer | 2007-2011 | −1.3* | 2011-2017 | −2.7* | −2.1* | [−2.3 to −2.0] |
| | Heart diseases | 2007-2017 | −1.0* | | | −1.0* | [−1.2 to −0.8] |
| | Cerebrovascular diseases | 2007-2013 | −3.1* | 2013-2017 | −1.5* | −2.5* | [−3.0 to −1.9] |
| | Pneumonia | 2007-2017 | −0.9* | | | −0.9* | [−1.2 to −0.7] |
| | Senility | 2007-2012 | −1.1* | 2012-2017 | −4.3* | −2.7* | [−2.9 to −2.4] |

* Significantly different from zero (p<0.05). LTC: long-term care, APC: annual percent change.
